# Avocado consumption is associated with better diet quality and nutrient intake, and lower metabolic syndrome risk in US adults: results from the National Health and Nutrition Examination Survey (NHANES) 2001–2008

**DOI:** 10.1186/1475-2891-12-1

**Published:** 2013-01-02

**Authors:** Victor L Fulgoni, Mark Dreher, Adrienne J Davenport

**Affiliations:** 1 Nutrition Impact, LLC, 9725 D Drive North, Battle Creek, MI, 49014, USA; 2 Nutrition Science Solutions, LLC, 900 S. Rainbow Ranch Rd, Wimberley, TX, 78676, USA; 3 3651 Toles Rd, Mason, MI, 48854, USA

**Keywords:** Avocado, Persea americana, Persea americana mill, Metabolic syndrome, Nutrient intake, BMI, Antioxidant

## Abstract

**Background:**

Avocados contain monounsaturated fatty acids (MUFA) dietary fiber, essential nutrients and phytochemicals. However, no epidemiologic data exist on their effects on diet quality, weight management and other metabolic disease risk factors. The objective of this research was to investigate the relationships between avocado consumption and overall diet quality, energy and nutrient intakes, physiological indicators of health, and risk of metabolic syndrome.

**Methods:**

Avocado consumption and nutrition data were based on 24-hour dietary recalls collected by trained NHANES interviewers using the USDA Automated Multiple Pass Method (AMPM). Physiological data were collected from physical examinations conducted in NHANES Mobile Examination Centers. Diet quality was calculated using the USDA’s Healthy Eating Index-2005. Subjects included 17,567 US adults  ≥ 19 years of age (49% female), including 347 avocado consumers (50% female), examined in NHANES 2001–2008. Least square means, standard errors, and ANOVA were determined using appropriate sample weights, with adjustments for age, gender, ethnicity, and other covariates depending on dependent variable of interest.

**Results:**

Avocado consumers had significantly higher intakes of vegetables (p < 0.05); fruit, diet quality, total fat, monounsaturated and polyunsaturated fats, dietary fiber, vitamins E, K, magnesium, and potassium (p < 0.0001); vitamin K (p = 0.0013); and lower intakes of added sugars (p < 0.0001). No significant differences were seen in calorie or sodium intakes. Body weight, BMI, and waist circumference were significantly lower (p < 0.01), and HDL-C was higher (p < 0.01) in avocado consumers. The odds ratio for metabolic syndrome was 50% (95th CI: 0.32-0.72) lower in avocado consumers vs. non-consumers.

**Conclusions:**

Avocado consumption is associated with improved overall diet quality, nutrient intake, and reduced risk of metabolic syndrome. Dietitians should be aware of the beneficial associations between avocado intake, diet and health when making dietary recommendations.

## Background

Dietary guidelines around the world recommend increased consumption of fruit and vegetables because they have low-to-medium energy density and are important contributors of major shortfall nutrients including dietary fiber, vitamins A, C and K, magnesium, and potassium [1,2]. Fruit and vegetables contain a diverse mixture of phytochemicals that may help support health and wellness and potentially reduce the risk of chronic diseases [3]. Data indicate that in the US, <3% of men and <6% of women aged 19 to 50 years consume the number of daily fruit and vegetable servings recommended [4,5].

Hass avocado (*Persea americana*) is a medium-size fruit with a pleasant, creamy, smooth texture. About 90% of the avocados consumed in the US and a majority of avocados worldwide are Hass avocados [5]. The avocado is a medium energy dense (1.7 kcal/g) fruit because it contains about 80% water and dietary fiber. Unlike other fruits, avocados are low in sugar and contain 15% MUFA rich oil, which helps to increase the bioavailability of carotenoids from salads and salsa often consumed with avocados [5-8]. Avocados also contain a variety of vitamins, minerals and phytochemicals such as lutein, phenolic antioxidants, and phytosterols associated with numerous potential health benefits [5,7,8]. Although there are 8 preliminary clinical studies on avocado and cardiovascular health [9-16], research on avocado health benefits is limited. The purpose of this study was to for the first time investigate associations between avocado consumption and diet quality, energy and nutrient intakes, body weight and metabolic syndrome risk factors in a nationally representative sample of US adults.

## Methods

NHANES is an ongoing initiative conducted by the National Center for Health Statistics of the Centers for Disease Control and Prevention to collect information on the health and nutritional status of a nationally represented cross-sectional sample of the total civilian, noninstitutionalized US population. The NHANES design is a stratified, multistage probability sample based on selection of counties, blocks, households, and the number of people within households. In 1999, the survey became a continuous program with a changing focus on a variety of pertinent health and nutrition measurements [17]. The methods and study design for NHANES have been previously described [18,19]. Data from 24-hour dietary recalls were collected using the Automated Multiple-Pass Method (AMPM) [20]. The Day 1 interview was conducted in person in a Mobile Examination Center (MEC) and was used for this study.

Energy and nutrient intakes were calculated using the United States Department of Agriculture (USDA) Food and Nutrient Database for Dietary Studies (FNDDS) versions 1.0 [21] 2.0 [22] and 3.0 [23]. NHANES uses the FNDDS to process and analyze dietary recall data. These databases have been previously described [24].

We used the MyPyramid Equivalents Database (MPED) versions 1.0 [25] and 2.0 [26] to examine consumption in terms of MyPyramid now called MyPlate [27] servings (hand matching foods without values in more recent NHANES surveys). The MPED translates dietary recall data into equivalent servings of the seven MyPlate major food groups and corresponding subgroups. The number of MyPlate servings was based on the 24-hr food dietary recall data from NHANES 2001–2008 participants. Avocado consumers were identified as NHANES 2001–2008 participants who reported eating any amount of avocado during the 24-hour dietary recall.

Diet quality was calculated using the USDA’s Healthy Eating Index (HEI)-2005 [28-30]. The HEI-2005 is a measure of diet quality that indicates how closely diets adhere to the 2005 Dietary Guidelines for Americans, and is primarily used by the USDA to monitor the diet quality of the US population. The original HEI was created in 1995 and revised in 2006 to reflect the 2005 Dietary Guidelines for Americans. Food group standards [31] and the development and evaluation of the HEI-2005 have been previously described [32,33].

Health indices evaluated included body weight, body mass index (BMI), waist circumference, HDL cholesterol (HDL-C), and risk of metabolic syndrome. The latter was defined as the presence of three or more of the following components: waist circumference ≥ 40 in (102 cm) for males or ≥ 35 in (88 cm) for females; triglycerides ≥ 150 mg/dL; HDL-C <40 mg/dL for males and < 50 mg/dL for females; blood pressure ≥ 130/85 mm Hg; or fasting glucose ≥ 100 mg/dL [34].

Least square means, standard errors of the mean, and ANOVA were determined for avocado consumption, diet quality, energy and nutrient intakes, and physiological markers of metabolic disease risk (ie, body weight, BMI, waist circumference, and HDL-C) in avocado consumers and non-consumers. The results were weighted using the NHANES examination sample weights to produce national estimates and adjust for the complex sample design of NHANES. Diet quality and food group/nutrient intakes were adjusted for age, gender, ethnicity, poverty income ratio, self-reported physical activity level, smoking status, alcohol intake, and energy intake. Physiological variables were adjusted for age, gender, ethnicity, poverty income ratio, self-reported physical activity level, smoking status, alcohol intake and BMI (for non-weight related variables). Minimal statistical significance was set at p < 0.05. Data were analyzed with the statistical packages SAS version 9.2 (SAS Institute, Cary, NC) and SUDAAN version 10.0.1 (2009, RTI, Research Triangle Park, NC).

## Results

The NHANES 2001–2008 sample included 17,567 individuals ≥ 19 years of age (49% female). Data indicate that approximately 2% of individuals were avocado consumers (n = 347; 50% female). Mean avocado intake (Table 1) was 70.1 ± 5.4 g/d (approximately one half of a medium-sized fruit), which contains about 114 calories of which 95 calories come from fat [5,35]. Consumption of avocados was higher in males than females.

**Table 1:** Average daily avocado consumption by gender^1,2^

	**Avocado Consumption**
	**(g/d)**
Total (n = 347)	70.1 ± 5.4
Males (n = 174)	75.3 ± 6.3
Females (n = 173)	66.7 ± 7.3

^1^Data Source: NHANES 2001–2008; analyses adjusted for complex sample design and using sample weights.

^2^Least square mean ± standard error of the mean.

Compared to non-consumers, diet quality (Table 2) was significantly (p < 0.0001) higher in avocado consumers. The latter had significantly (p < 0.05) higher intakes of fruit and vegetables and lower intake of added sugars. There were no significant differences between consumers and non-consumers for intakes of total grains, whole grains, dairy, meat and beans, and discretionary fats (as oil or solid fat).

**Table 2:** Diet quality and average daily serving intakes of MyPlate food groups for avocado consumers and non-consumers^1^

**Food group**	**Consumers (n = 347)**	**Non-consumers (n = 17,220)**	**p-value**
Healthy Eating Index - 2005	57.1 ± 1.5^2^	50.8 ± 0.2	0.0001
Fruits (cup equiv./d)	1.6 ± 0.1	1.0 ± 0.02	< 0.0001
Vegetables (cup equiv./d)	1.8 ± 0.1	1.6 ± 0.02	< 0.05
Total Grains (oz equiv./d)	6.8 ± 0.04	6.8 ± 0.04	n.s.^3^
Whole Grains (oz equiv./d)	0.7 ± 0.09	0.7 ± 0.02	n.s.
Dairy (cup equiv./d)	1.6 ± 0.1	1.6 ± 0.02	n.s.
Meat and Beans (oz equiv./d)	5.9 ± 0.3	6.1 ± 0.1	n.s.
Added sugar (tsp/d)	15.7 ± 0.9	19.7 ± 0.3	< 0.0001
Discretionary fat, Oils (g/d)	21.0 ± 1.3	19.0 ± 0.2	n.s.
Discretionary fat, Solid (g/d)	46.8 ± 0.2	43.4 ± 1.8	n.s.

^1^Data Source: NHANES 2001–2008, n = 17,567; analyses adjusted for complex sample design using sample weights with age, gender, ethnicity, poverty income ratio, self-reported physical activity level, smoking status, alcohol intake, and energy intake as covariates.

^2^Least square mean ± standard error of the mean.

^3^n.s.: not significant.

Compared with non-consumers, those who ate avocados (Table 3) had significantly (p < 0.0001) higher intakes of total fat, monounsaturated fat, polyunsaturated fat, dietary fiber, vitamin E, magnesium and potassium, and vitamin K (p = 0.0013). Avocado consumers had significantly lower carbohydrate (p < 0.001). No significant differences were seen in calorie or sodium intakes. Those who ate avocados had significantly (p < 0.01) lower body weight, BMI, and smaller waist circumference (Table 4). HDL-C was significantly (p < 0.01) higher in avocado consumers compared to non-consumers. The odds ratio for metabolic syndrome was 50% lower in those who ate avocados compared to those who did not (95% confidence interval [CI]: 0.34, 0.72) .

**Table 3:** Average daily nutrient intakes for avocado consumers and non-consumers^1^

**Component**	**Consumers (n = 347)**	**Non-consumers (n = 17,220)**	**p-value**
Calories, kcal	2250 ± 65^2^	2190 ± 11	n.s.^3^
Total fats (g)	92.0 ± 1.6	82.9 ± 0.3	<0.0001
Monounsaturated fat (g)	36.3 ± 0.6	30.7 ± 0.1	<0.0001
Polyunsaturated fat (g)	19.5 ± 0.5	17.4 ± 0.1	<0.0001
Carbohydrates (g)	250 ± 4.2	266 ± 0.9	<0.001
Dietary fiber (g)	21.5 ± 0.8	15.8 ± 0.2	<0.0001
Vitamin E, as α-tocopherol (mg)	9.0 ± 0.4	7.3 ± 0.1	0.0001
Vitamin K, μg	141 ± 13	95.5 ± 1.9	0.0013
Magnesium (mg)	328 ± 7.9	290 ± 1.8	<0.0001
Potassium (mg)	3133 ± 56.9	2710 ±13.7	<0.0001
Sodium (mg)	3359 ± 67.6	3491 ±12.3	n.s.

^1^Data Source: NHANES 2001–2008, n = 17,567; analyses adjusted for complex sample design using sample weights with age, gender, ethnicity, poverty income ratio, self-reported physical activity level, smoking status, alcohol intake, and energy intake (except for calories) as covariates.

^2^Least square mean ± standard error of the mean.

^3^n.s.: not significant.

**Table 4:** Physiological measures of avocado consumers and non-consumers^1^

**Indices**	**Consumer**	**Non-consumer**	**p-value**
Body weight (kg)	78 ± 1.1^2^	81.4 ± 0.3	<0.01
BMI (kg/m^2^)	26.7 ± 0.4	28.4 ± 0.1	<0.0001
Waist circumference (cm)	93.2 ± 0.8	97.2 ± 0.2	<0.0001
HDL-cholesterol (mg/d)	55.0 ± 0.8	52.6 ± 0.2	<0.01

^1^Data Source: NHANES 2001–2008, n = 17,567; analyses adjusted for complex sample design and using sample weights with age, gender, ethnicity, poverty income ratio, self-reported physical activity level, smoking status, alcohol intake, and body mass index (for HDL-cholesterol only).

^2^Least square mean ± standard error of the mean.

## Discussion

This is the first report to investigate avocado consumption among the US population ≥ 19 years of age, and explore its relationships to diet quality, energy and nutrient intakes, and physiological markers of health. In this report, avocado consumption was associated with significant differences in diet quality and nutrient intakes, higher HDL-C levels, and lower body weight, BMI, waist circumference, and risk of metabolic syndrome. The improved diet quality, nutrient intake, and HDL-C outcomes associated with avocado consumption are consistent with the avocado composition and clinical data [5,9-16]. Since energy intake was not different between consumers and nonconsumers, the reported lower body weight, BMI and waist circumference for the avocado consumers needs to be further investigated. The effect of diets on weight control may be dependent of a number of factors such as energy density, macronutrient bioavailability, and potentially other factors such as food physical properties [36-38]. Epidemiological evidence and intervention studies generally support the association between the consumption of energy-dense/high-fat diets with being overweight [36,37]. Insulin resistance, a key pathogenic link underlying the cluster of metabolic abnormalities seen in metabolic syndrome, is adversely effected by saturated fat and improved with MUFA [39-41]. Tree nuts, which are similar to avocado in dry weight composition including dietary fiber and MUFA content, have not been shown to increase body weight or metabolic syndrome risk in numerous clinical and epidemiological studies [42-46].

Epidemiologic studies dating back to the 1970s show the health benefits of MUFAs [45]. The Seven Countries Study found that death rates were positively related to the average percentage of energy from saturated fatty acids (SFAs) and negatively related to the percentage of dietary energy from MUFAs [47]. Prospective studies of US nurses have demonstrated an estimated reduction of 19% in the risk of coronary disease when the intake of MUFAs is increased by 5% (as a percentage of total energy intake) [48]. More recently, Moreno et al. [49] found that a MUFA-rich diet may have favorable effects on cardiovascular risk by preventing the oxidative modifications of LDL-C and reducing macrophage uptake of plasma oxidized LDL. Studies also suggest that MUFAs may have a modest antihypertensive effect and could improve insulin sensitivity [40].

Results of this study indicate avocado consumption is associated with improved nutrient intakes including higher intake of mono- and polyunsaturated fat, dietary fiber and several vitamins and minerals; lower body weight, BMI, and waist circumference; higher HDL-C; and decreased risk of metabolic syndrome. These findings suggest a role for avocados in improving dietary quality and possibly reducing the risk of metabolic syndrome in the United States. Further research is needed to verify this epidemiological data and study the potential association between increased intake of avocados and other dietary components.

### Limitations of the study

Energy and food group/nutrient intakes, including HEI-2005 scores, in this study were based on single 24-hour dietary recalls. While dietary recalls were collected via AMPM, the best methodology available, there are still limitations to this dietary collection method [24]. Recalls may be inaccurate and biased due to misreporting or memory lapses. Even though the AMPM method has been validated against weighed food records, energy can be underestimated by 3% for a population of normal weight and up to 11% for overweight and obese persons [18,24].

The present report comes from cross-sectional epidemiological data and thus cannot provide causal evidence between avocado consumption and improvements in diet quality, nutrient intakes, body weight, BMI, waist circumference, or indices of health. The number of avocado consumers was relatively small and while we used numerous covariates in an attempt to remove effects of other variables, residual confounding may still exist. The associations reported here should be interpreted accordingly.

## Conclusions

This study is the first to explore associations between avocado consumption and diet quality, nutrient and energy intakes, and metabolic disease risk factors in a stratified random sample of the total civilian, noninstitutionalized US population. Use of NHANES estimates makes it possible to generalize findings to the population at large. The data demonstrate significant associations between avocado consumption and a higher HEI diet quality score and nutrient intakes; lower body weight, BMI, and waist circumference; higher HDL-C levels; and reduced risk of metabolic syndrome. Dietitians can recommend consumption of avocados as part of a healthful diet that focuses on increased fruit and vegetable intake. Avocados can be incorporated into the diets of most adults, and may be of additional benefit to those who have increased risk for metabolic disease risk factors.

This study was supported by the Hass Avocado Board.

## Competing interests

VLF as Senior Vice President of Nutrition Impact, LLC performs consulting and database analyses for various food and beverage companies and related entities. MD is a nutrition science consultant for various food companies and related entities including the Hass Avocado Board. AJD is an analyst for School Nutrition Programs within the Michigan Department of Education and also serves as a freelance nutrition consultant.

## Authors’ contributions

VLF designed the study, was primarily responsible for the data analysis, and provided critical input into the manuscript; MD and AJD helped with data analysis and drafted the manuscript. All authors read and approved the final manuscript.
